# Reinforcement learning for UAV flight controls: Evaluating continuous space reinforcement learning algorithms for fixed-wing UAVs

**DOI:** 10.1371/journal.pone.0334219

**Published:** 2025-10-09

**Authors:** Hasan Raza Khanzada, Adnan Maqsood, Abdul Basit

**Affiliations:** 1 School of Interdisciplinary Engineering and Sciences (SINES), National University of Science and Technology, Islamabad, Pakistan; 2 Department of Electrical Engineering, Pakistan Institute of Engineering and Applied Sciences (PIEAS), Islamabad, Pakistan; Van Lang University: Truong Dai hoc Van Lang, VIET NAM

## Abstract

Flight controls are experiencing a major shift with the integration of reinforcement learning (RL). Recent studies have demonstrated the potential of RL to deliver robust and precise control across diverse applications, including the flight control of fixed-wing unmanned aerial vehicles (UAVs). However, a critical gap persists in the rigorous evaluation and comparative analysis of leading continuous-space RL algorithms. This paper aims to provide a comparative analysis of RL-driven flight control systems for fixed-wing UAVs in dynamic and uncertain environments. Five prominent RL algorithms that include Deep Deterministic Policy Gradient (DDPG), Twin Delayed Deep Deterministic Policy Gradient (TD3), Proximal Policy Optimization (PPO), Trust Region Policy Optimization (TRPO) and Soft Actor-Critic (SAC) are evaluated to determine their suitability for complex UAV flight dynamics, while highlighting their relative strengths and limitations. All the RL agents are trained in a same high fidelity simulation environment to control pitch, roll and heading of the UAV under varying flight conditions. The results demonstrate that RL algorithms outperformed the classical PID controllers in terms of stability, responsiveness and robustness, especially during environmental disturbances such as wind gusts. The comparative analysis reveals that the SAC algorithm achieves convergence in 400 episodes and maintains a steady-state error below 3%, offering the best trade-off among the evaluated RL algorithms. This analysis aims to provide valuable insight for the selection of suitable RL algorithm and their practical integration into modern UAV control systems.

## 1 Introduction

Unmanned Aerial Vehicles (UAVs) have gained significant research attention in recent years with numerous reported applications in navigation, environmental monitoring, package delivery, precision agriculture and disaster management [1,2]. In particular, fixed-wing UAVs offer several advantages over rotary wing UAVs, including longer flight endurance, higher speed, greater range, and better efficiency over long distances [3]. Although they offer distinct advantages but controlling and maintaining them to ensure stable flight remains a significant challenge due to their complex and non-linear dynamics [4].

In the existing literature, Proportional Integral Derivative (PID) controllers have been commonly used for UAV control owing to simpler design and reliable performance [5]. These controllers continuously compute the error between the commanded and detected flight parameters and try to correct them using feedback loops [6]. Although PID controllers are simple and widely used, they struggle with the complex and unpredictable nature of UAV operations. The performance of traditional PID control mechanisms degrades under model uncertainty, dynamic coupling, and non-linear interactions between flight variables, such as coupled pitch-roll dynamics [7]. Another major limitation of classical control techniques is the requirement for accurate mathematical models of UAV dynamics. These models are often difficult to obtain and may not include real-world dynamics such as sudden wind gusts and uneven payload distribution. Thus, there is a need to further explore more adaptive and intelligent control strategies for fixed-wing UAVs [8].

In recent years, Reinforcement learning (RL) has emerged as a promising solution for dealing with the complex UAV dynamics. Despite some notable progress, the existing RL-based UAV control studies often lack a unified evaluation framework. Moreover, the existing investigations have adopted different flight configurations and environmental assumptions, which makes them difficult to compare. This study aims to systematically compare the performance, architectures, and training methodologies of multiple RL approaches to provide practical insight into the strengths and limitations of RL-based UAV control strategies. The study also provides key considerations for further research, particularly in the optimization of RL strategies for flight controls. By evaluating different RL algorithms under realistic flight conditions, this work provides a foundation for the development of intelligent and adaptive control systems. This evaluation can contribute to the enhancement of UAV performance in dynamic and uncertain environments. Accordingly, a systematic comparative analysis is conducted for RL-based flight control algorithms for fixed-wing UAVs under consistent and realistic flight scenarios. The main contributions of this work are as follows:

1. **Unified Evaluation Framework:** Development of a simulation framework that integrates a high-fidelity 6-DOF fixed wing UAV model with reinforcement learning-based flight controllers, enabling consistent evaluation across all control axes.
2. **Comprehensive Algorithm Benchmarking:** Implementation and in-depth evaluation of five state-of-the-art continuous control RL algorithms (DDPG, TD3, PPO, TRPO, and SAC) across key UAV control tasks including roll, pitch, yaw, altitude, airspeed, and heading regulation.
3. **Comparison Against Classical Control:** Quantitative comparison of RL controllers with conventional PID controllers under dynamic environmental conditions, highlighting improvements in stability, responsiveness, and disturbance rejection.
4. **Systematic Hyperparameter Tuning:** Systematic documentation of hyperparameter tuning and training settings to ensure reproducibility and facilitate practical deployment.
5. **Guidance on Algorithm Selection:** Practical guidance for selecting suitable RL algorithms for real-time UAV flight control applications.

The paper starts with an Introduction (Sect 1) followed by a detailed review of Related Work (Sect 2) that entails recent advancements in RL for UAV flight control systems. Sect 3 provides a comprehensive description of the system and discusses the airframe and aerodynamic modeling, and the guidance and control framework. In Sect 4, the RL algorithms and their implementation details are presented. The training and evaluation processes for different RL schemes are described in Sect 5, and Sect 6 presents the comparative performance analysis of RL algorithms and PID controllers. The results are discussed in Sect 7, while the key findings are described in Sect 8. Finally, Sect 9 concludes the paper.

## 2 Related work

Recent years have witnessed growing interest in applying reinforcement learning (RL) to UAV flight control, particularly for managing non-linear dynamics, coupling between flight parameters, and external disturbances that challenge traditional PID-based methods. Several studies have validated RL’s capacity to maintain stable flight performance under conditions such as wind gusts and payload variations, often outperforming classical controllers in both simulation and real-world tests [9,10].

Early investigations into reinforcement learning applications for aircraft control such as pitch control laid the groundwork for managing complex flight dynamics. Jiang and Kamel’s [11] seminal work demonstrated the viability of RL algorithms for critical aircraft control tasks like pitch stabilization, establishing a historical context for RL in aerospace. More recently, Din et al. [12] emphasized the benefits of model-free, data-driven approaches for enhancing RL’s adaptability in challenging UAV environments.

Several studies have shown the potential of RL in UAV control tasks. Zahmatkesh et al. [9] proposed an improved Q-learning algorithm for robust attitude control of an agile aircraft. Their method applied dynamic state weighting to stabilize the aircraft during aggressive maneuvers. Kimathi [13] implemented a Deep Q-Network (DQN)-based heading controller on a fixed-wing UAV. Using the X-Plane simulator, the trained policy successfully managed lateral navigation under moderate wind conditions.

Expanding on directional control, comprehensive studies have explored the direct application of RL for fixed-wing UAV heading control. These works confirmed its real-world effectiveness through simulation environments like the aforementioned X-Plane. Kimathi, Kang’ethe, and Kihato [14] detailed the successful implementation of RL for heading control in fixed-wing UAVs within a realistic simulation environment. Their findings reinforced the practicality of RL for precise navigational tasks. Zhen et al. [10] employed Proximal Policy Optimization (PPO) for end-to-end attitude control of fixed-wing UAVs. Their model-free RL framework directly mapped observations to control outputs without relying on aerodynamic models. The controller was trained entirely in simulation and demonstrated the ability to track pitch, roll, and yaw commands in the presence of wind disturbances and measurement noise. The agent’s performance was validated in simulation, showcasing the potential of deep RL for full three-axis flight control.

Beyond individual control axes, deep reinforcement learning (DRL) has shown significant promise in enabling complex path-following behaviors for UAVs. Zhang et al. [15] highlighted the advanced capabilities of DRL for managing complex UAV trajectories. In a different domain, Wang et al. [16] developed a vision-based DRL controller for autonomous obstacle avoidance. Using depth images and an actor-critic architecture, their method enabled quadrotors to avoid static and dynamic obstacles in complex indoor and outdoor environments. Their results showed DRL can manage real-time navigation without explicit mapping or localization. Moreover, DRL’s potential extends to ensuring stability under external forces. Several studies have specifically investigated active wind rejection across different UAV types. Xing et al. [17] showed that RL can generate robust strategies to counter unpredictable disturbances such as wind, ensuring reliable UAV operation. Similarly, Wang et al. [18] highlighted the importance of adaptive and fault-tolerant control for handling model uncertainties and system faults. This represents a key advantage that RL offers over traditional methods in uncertain and impaired operational conditions.

In addition to these task-specific applications, recent work has focused on RL algorithms designed for continuous control. Algorithms such as Deep Deterministic Policy Gradient (DDPG), Twin Delayed Deep Deterministic Policy Gradient (TD3), Proximal Policy Optimization (PPO), Trust Region Policy Optimization (TRPO), and Soft Actor-Critic (SAC) have been successfully applied to various UAV control problems. Each offers unique advantages. DDPG extends Q-learning to continuous action spaces via actor-critic structures [19]. TD3 improves upon DDPG by introducing twin critics and delayed policy updates to mitigate overestimation bias [20]. PPO and TRPO enhance training stability using clipped surrogate objectives and trust region constraints, respectively [21,22]. While SAC introduces entropy regularization for improved robustness and exploration in stochastic environments [23].

For fixed-wing UAVs, DRL algorithms, including DDPG, have been specifically applied to attitude control and altitude-hold strategies. These applications demonstrated their capacity for precise maneuvers. Li et al. [24] expanded on this by demonstrating the effectiveness of DRL in fixed-wing attitude control. These algorithms also support complex behaviors such as UAV formation flight. Xu et al. [25] demonstrated this by applying PPO to multi-agent coordination. The utility of continuous-space DRL extends across various aerial platforms, including multi-copters, for fine-grained control. Manukyan et al. [26] extended the scope of DRL’s application to continuous control in other UAV types. Their work underscored the generalizability of DRL algorithms for precise flight management. Likewise, enhanced SAC variants have enabled 3D trajectory planning in dynamic environments. Zhou et al. [27] demonstrated its capability for real-time navigation and flight management.

These algorithms have been studied in isolation across varied UAV applications. For instance, Bøhn et al. [28] applied PPO for trajectory tracking of a quadrotor, achieving smooth path following and better generalization compared to PID control. Hu et al. [29] extended PPO to multi-agent UAV formations for coordinated tracking under time-varying conditions. Hwang et al. [30] proposed a piecewise formulated reward for fixed-wing UAV tasks, combining terminal success or failure rewards with per-step distance feedback to guide incremental training under complex flight dynamics. Kong et al. [31] introduced an actor-critic-based adaptive flight controller that stabilized aircraft under fault conditions and demonstrated its resilience to actuator and sensor anomalies.

In addition to these algorithmic studies, recent work has explored model-free reinforcement learning approaches specifically designed to address nonlinearities, uncertainties, and under actuation in aerial and robotic systems. Abouheaf et al. [32] developed an online adaptive RL controller for flexible-wing UAVs that handled actuator nonlinearities and modeling uncertainties without relying on prior knowledge of system dynamics. Tutsoy et al. [33] proposed a model-free adaptive control strategy for underactuated manipulators exhibiting chaotic dynamics, demonstrating robustness in uncertain environments. In the context of emergency UAV operation, Tutsoy et al. [34] also proposed bio-inspired, model-free path planning strategies for faulty UAVs under actuator impairments and parametric uncertainties. In UAV contexts, Olivares et al. [35] compared model-free and model-based RL for fixed wing attitude control under varying wind disturbances, showing superior generalization capabilities for model-free agents. Furthermore, methods that integrate prior knowledge into reinforcement learning path planning algorithms can enhance performance, suggesting avenues for making RL-based systems more robust. Shi et al. [36] provides insight into strategies for enhancing RL performance by incorporating external information, which is relevant for improving the robustness and efficiency of control policies in complex and uncertain environments. This broader context also includes the autonomous and cooperative control of UAV clusters, which heavily relies on multi-agent reinforcement learning principles. Xu and Chen [37] directly addresses the application of multi-agent RL in coordinating multiple UAVs, underscoring the necessity of robust and scalable learning algorithms for complex swarm behaviors.

Building on these prior efforts, recent advancements in deep RL have expanded the scope of UAV applications, from basic stabilization to complex navigation and coordination tasks. However, most studies emphasize specific tasks or algorithmic innovations without broader comparative assessments. This paper builds upon these foundations by organizing and evaluating diverse RL strategies within a comprehensive experimental framework, offering a clearer understanding of algorithmic trade-offs and deployment considerations across UAV control domains.

## 3 System description

**Fig 1:**
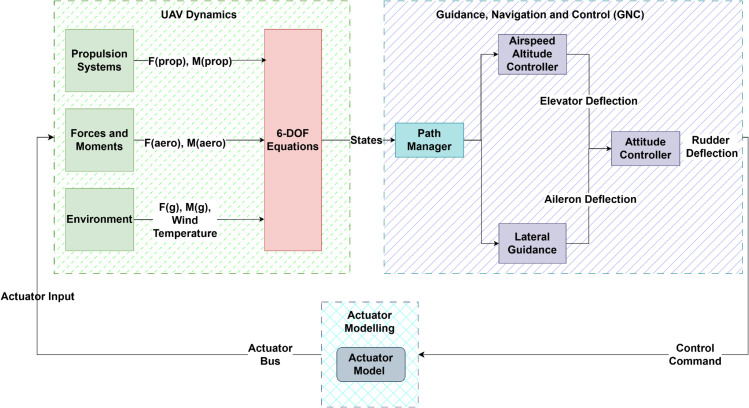
Modeling of a fixed wing UAV.

The UAV model considered in this investigation is a small fixed-wing aircraft, with the primary control surfaces being the elevator, which controls pitch and subsequently altitude, the aileron responsible for roll, and the rudder manages yaw [38]. The motion of a UAV is characterized by non-linear six degree of freedom (6-DOF) equations, which capture its behavior in three dimensional space under aerodynamic forces and moments [39]. The model is defined in a continuous action space, where variations in control inputs applied through control surface deflections directly regulate flight variables such as altitude and heading, as shown in Fig 1.

### 3.1 Airframe and aerodynamic modeling

The dynamics of the UAV are formulated through a set of mathematical expressions that incorporate both linear and angular accelerations [40]. The linear acceleration components, $\left(\dot{u},\dot{v},\dot{w}\right)$, are defined as:

$$\dot{u}=\frac{F_{x}}{m}-qw+rv-g\sin\theta$$ (1)

$$\dot{v}=\frac{F_{y}}{m}-pw+ru-g\cos\theta \sin\phi$$ (2)

$$\dot{w}=\frac{F_{z}}{m}-pv+qu-g\cos\theta \cos\phi$$ (3)

The angular acceleration components $\left(\dot{p},\dot{q},\dot{r}\right)$, are expressed as:

$$\dot{p}=\frac{1}{I_{xx}}\;(L-(I_{zz}-I_{yy})qr)$$ (4)

$$\dot{q}=\frac{1}{I_{yy}}\;(M-(I_{xx}-I_{zz})pr)$$ (5)

$$\dot{r}=\frac{1}{I_{zz}}\;(N-(I_{yy}-I_{xx})pq)$$ (6)

In Eqs (1)–(6), *u*,*v*, and *w* are the linear velocities, *p*,*q*, and *r* are the angular rates, $F_{x},F_{y}$, and *F*_*z*_ are the force components, *L*,*M*, and *N* are the moment components, $I_{x}x,I_{y}y$, and *I*_*z*_*z* are the moments of inertia, *m* is mass, and *g* is the gravitational acceleration [13].

### 3.2 Uncertainty modeling and simulation assumptions

The simulation environment introduces various sources of uncertainty to reflect the complexities encountered in real-world UAV operations. These uncertainties are categorized as internal or external, and as parametric or non-parametric, depending on their origin and modeling approach. Internally, UAV dynamics are modeled using full non-linear six-degree-of-freedom (6-DOF) rigid-body equations of motion, preserving the coupling between translational and rotational states. Aerodynamic forces and moments are computed using multidimensional lookup tables based on variables such as angle of attack, Mach number, and altitude. Control surface actuation is represented via second-order dynamics with saturation limits, incorporating realistic actuator lag and response constraints. Sensor uncertainty is modeled by injecting Gaussian noise into key measurements, including barometric pressure and altitude. In addition, fixed transport delays are introduced to mimic basic latency from onboard computation or communication channels. Externally, the simulation includes randomized disturbances to introduce non-deterministic flight conditions across training episodes. These include variations in initial conditions and atmospheric properties, ensuring that the agent learns in a dynamic and partially unpredictable environment. A detailed discussion of environmental disturbances is provided in Sect 3.3. Some real-world uncertainty sources remain unmodeled in the current software-in-the-loop (SIL) setup. These include structural flexibility, GNSS outages, wind shear gradients, hardware failures (e.g., servo malfunctions), and complex environmental factors such as rain, icing, or ground effect. While these are not explicitly represented, the variability introduced by actuator constraints, sensor noise, and environmental randomness still provides a sufficiently challenging learning environment. These limitations underscore the importance of future extensions involving hardware-in-the-loop (HIL) testing or real-world validation to better capture the full spectrum of operational uncertainties.

### 3.3 Environmental disturbances

The simulation introduces environmental noises because it is important to estimate the level of robustness of RL agents when exposed to them. Every RL agent is required to understand the operation of UAVs, even when faced with environmental uncertainty, including wind gusts and changes in air density [17].

- **Wind gust:** The simulation incorporates environmental disturbances, as evaluating the robustness of RL agents under such conditions is crucial. Each RL agent must learn to control the UAV despite encountering unforeseen environmental challenges, such as wind gusts and changes in air density [18].
- **Air density:** Air density variations with altitude were introduced to assess UAV performance under different atmospheric conditions. The air density has a notable effect on lift and drag forces critical for stable flight. In this paper, we have used a lapse rate of 0.0065 K / m, a sea level value of 1.225 kg/m^3^ for air density, and 288.15 K for temperature [41].

### 3.4 Flight profile

**Fig 2:**
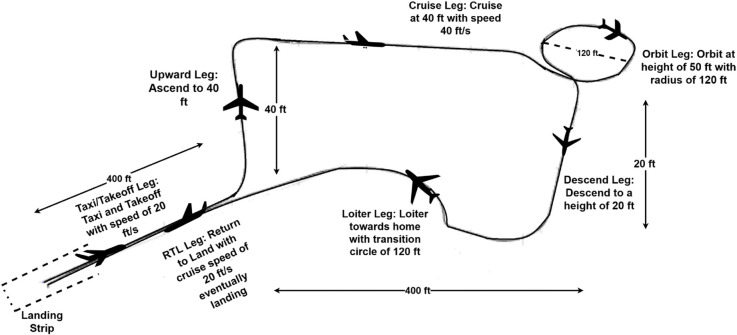
Track followed by fixed wing UAV.

In this paper, we have considered all the important segments of the flight to make the investigation more realistic. The adopted flight profile includes taxi, take-off, orbit, waypoint following, and landing. Each segment has been designed with specific parameters to ensure that the UAV follows a predefined path with desired altitude, heading, and other critical flight characteristics [15]. The flight track is depicted in Fig 2 with the constituent segments defined as.

1. **Taxi:** The mission commences with a ground roll at 20 ft/sec prior to take-off.
2. **Take-Off:** The UAV ascends at a rate of 3 ft/sec until reaching an altitude of 30 ft. During this phase, airspeed and pitch are carefully regulated to ensure a steady climb.
3. **Orbit:** At a designated waypoint, the UAV performs an orbital maneuver with a radius of 120 ft, completing a specified number of turns while maintaining constant altitude.
4. **Waypoint Navigation:** The UAV proceeds through sequential waypoints, each defined by particular altitude and heading requirements.
5. **Landing:** The mission concludes with a controlled descent at 3 ft/sec toward the runway, aligned with a *π* heading and a glide slope of $\frac{3\pi }{180}$.

### 3.5 Guidance and control system

The UAV control architecture is organized into nested loops, with an inner loop dedicated to stability and an outer loop responsible for waypoint tracking. The inner loop maintains aircraft stability by utilizing gyroscope and accelerometer measurements to implement rapid control surface corrections. In contrast, the outer loop regulates altitude and orientation by processing waypoint and navigation data, thereby enabling the UAV to follow predefined trajectories. This layered structure offers several benefits, including effective real-time management of flight dynamics, precise response to autopilot commands, and resilience against external disturbances such as wind gusts [42].

Complementing this, the guidance subsystem is designed to convert mission-level objectives into specific flight parameters, including altitude, airspeed, and attitude. Relying on path-planning algorithms, the guidance system autonomously adjusts flight profiles to reconcile operational objectives with dynamic environmental conditions [43].

#### 3.5.1 Altitude control.

The angular position of the nose with reference to the horizon, called pitch angle, can be varied by the deflection in the UAV elevator. The upward deflection causes the aircraft’s nose to pitch upward, increasing the angle of attack [44]. Whereas the downward deflection causes the UAV’s nose to pitch downward, reducing the angle of attack and therefore decreasing lift [11].

Mathematically, the change in vertical motion due to elevator deflection can be linked to the rate of change of pitch angle, which influences the altitude rate as discussed in Eq (7).

$$\dot{h}=V\sin(\theta )$$ (7)

**Fig 3:**
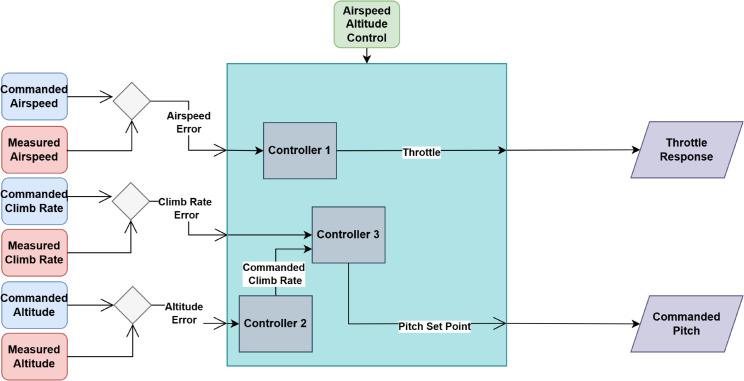
Working of airspeed and altitude controller.

Where $\dot{h}$ is the rate of altitude change, *V* is the airspeed, and *θ* is the pitch angle controlled by elevator deflection. Fig 3 illustrates how the pitch angle, controlled by elevator deflection, impacts both airspeed and altitude regulation in the system. Airspeed, Climb rate and Altitude errors are computed individually which is then used to find throttle response and pitch required to stay on track.

#### 3.5.2 Heading control.

**Fig 4:**
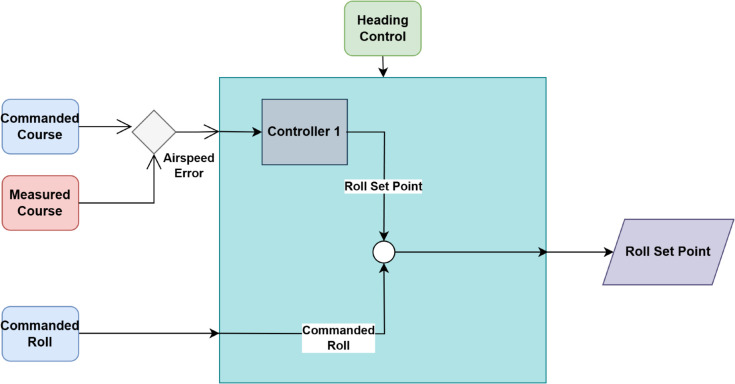
Working of heading controller.

The deflection of the rudder controls the direction of the UAV by altering the yaw angle, which is the rotation of the UAV around its vertical axis as illustrated in Fig 4. When the rudder is adjusted towards right, it causes the UAV’s nose to yaw to the right, shifting its heading in a clockwise direction. On the other hand, leftward deflection causes the UAV’s nose to yaw to the left, resulting in a counterclockwise change in heading. The change in horizontal direction due to rudder deflection can be mathematically linked to the rate of change of the yaw angle, which determines the UAV’s heading rate.

$$\dot{\psi }=\frac{V\sin(\phi )}{\cos(\theta )}$$ (8)

Where $\dot{\psi }$ is the rate of change of the heading angle, *V* is the airspeed, *ϕ* is the roll angle, which affects the turn rate, and *θ* is the pitch angle, which influences the effectiveness of the rudder during flight. The relationship discussed in Eq (8) highlights how rudder deflection influences the UAV’s heading by modulating the yaw angle, allowing it to change direction efficiently [45]. Smooth, accurate turning and track adjustments are made possible by the use of heading control within a coordinated flight strategy [14].

#### 3.5.3 Attitude controllers.

The attitude controllers are described in the subsequent paragraphs.

1. **Roll Controller**: The roll controller manages the UAV’s roll angle, which determines the tilt of the wings. By adjusting aileron deflection, the roll controller ensures that the UAV tilts to the desired roll angle, aiding in stable turns and coordinated maneuvers. The roll angle φ is critical for adjusting the aircraft’s lateral balance and is linked to the roll rate as represented below
  $$\dot{\phi }=f_{\phi }(p,\phi_{\text{trim}},C_{l},\delta_{a})=p+\frac{1}{J_{x}}(C_{l}\cdot \delta_{a}-\phi_{\text{trim}}),$$ (9)
  where $\phi_{\text{trim}}$ is the trim angle, *C*_*l*_ is the roll moment coefficient, $\delta_{a}$ is the aileron deflection, and *J*_*x*_ is the roll moment of inertia.
2. **Pitch Controller**: The pitch controller regulates the pitch angle *θ* of the UAV, controlling its nose position relative to the horizon. Altitude variations are achieved through the pitch controller, which regulates elevator deflections to command both climb and descent. The pitch angle *θ* plays a central role in maintaining the UAV’s longitudinal balance and is related to the pitch rate as:
  $$\dot{\theta }=f_{\theta }(q,\theta_{\text{trim}},C_{m},\delta_{e})=q+\frac{1}{J_{y}}(C_{m}\cdot \delta_{e}-\theta_{\text{trim}}),$$ (10)
  where $\theta_{\text{trim}}$ denotes the trim angle, *C*_*m*_ represents the pitching moment coefficient, $\delta_{e}$ corresponds to the elevator deflection, and *J*_*y*_ refers to the pitch moment of inertia.
3. **Yaw Controller**: The yaw controller regulates the UAV’s heading, or yaw angle *ψ*, which specifies the direction of flight. By actuating rudder deflections, it alters the aircraft’s orientation about the vertical axis, thereby enabling turning maneuvers. The yaw angle *ψ* is related to the yaw rate as follows:
  $$\dot{\psi }=f_{\psi }(r,\psi_{\text{trim}},C_{n},\delta_{r})=r+\frac{1}{J_{z}}(C_{n}\cdot \delta_{r}-\psi_{\text{trim}}),$$ (11)
  where $\psi_{\text{trim}}$ is the trim angle, *C*_*n*_ is the yaw moment coefficient, $\delta_{r}$ is the rudder deflection, and *J*_*z*_ is the yaw moment of inertia [4].

#### 3.5.4 Rate controllers.

**Fig 5:**
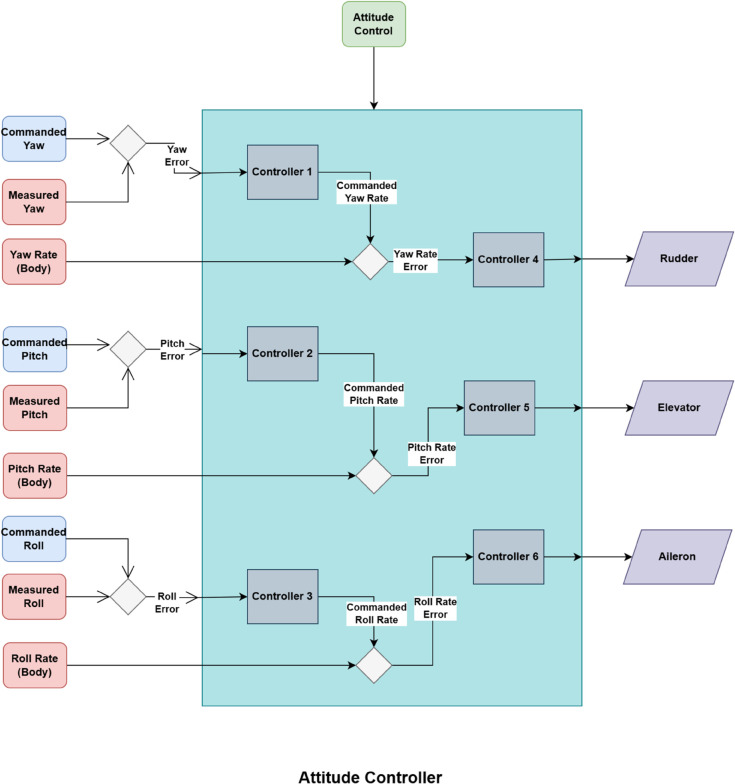
Working of attitude controller.

The rate controllers manage the rates of roll, pitch, and yaw to ensure that the UAV responds smoothly to attitude adjustments made by the roll, pitch, and yaw controllers. They act as stabilizing loops within the main attitude control loops. Fig 5 shows the working of the Attitude Controller managing roll, pitch and yaw rates. The rate controllers are delineated in the following paragraphs.

1. **Roll Rate Controller**: The roll rate controller directly controls the rate of roll *p*, which is essential for achieving the desired roll angle set by the roll controller. It modulates aileron input to maintain a stable and responsive roll motion as explained below
  $$p=K_{p_{\phi }}(\phi_{\text{desired}}-\phi_{\text{actual}})+K_{d_{\phi }}(\frac{d(\phi_{\text{desired}}-\phi_{\text{actual}})}{dt})+\frac{\phi_{\text{trim}}}{J_{x}},$$ (12)
  where $K_{p_{\phi }}$ is the proportional gain, and $\phi_{\text{desired}}$ and $\phi_{\text{actual}}$ are the desired and actual roll angles, respectively [46].
2. **Pitch Rate Controller**: The pitch rate controller governs the rate of pitch *q*, stabilizing the UAV’s pitch movement to match the pitch angle command from the pitch controller. Elevator adjustments by the pitch rate controller maintain smooth pitch changes as explained below
  $$q=K_{p_{\theta }}(\theta_{\text{desired}}-\theta_{\text{actual}})+K_{d_{\theta }}(\frac{d(\theta_{\text{desired}}-\theta_{\text{actual}})}{dt})+\frac{\theta_{\text{trim}}}{J_{y}},$$ (13)
  where $K_{p_{\theta }}$ is the proportional gain, and $\theta_{\text{desired}}$ and $\theta_{\text{actual}}$ are the desired and actual pitch angles, respectively.
3. **Yaw Rate Controller**: The Eq 14 explains how yaw rate controller controls the yaw rate *r*, thus helping the UAV achieve the target yaw angle set by the yaw controller . By adjusting rudder inputs, the yaw rate controller stabilizes heading changes as follows:
  $$r=K_{p_{\psi }}(\psi_{\text{desired}}-\psi_{\text{actual}})+K_{d_{\psi }}(\frac{d(\psi_{\text{desired}}-\psi_{\text{actual}})}{dt})+\frac{\psi_{\text{trim}}}{J_{z}},$$ (14)
  where $K_{p_{\psi }}$ is the proportional gain, and $\psi_{\text{desired}}$ and $\psi_{\text{actual}}$ are the desired and actual yaw angles, respectively.

These six individual controllers work in tandem to maintain a stable and accurate flight path, with the attitude controllers setting the desired orientations and the rate controllers ensuring smooth transitions and stability [47].

## 4 RL in flight controls

### 4.1 Reinforcement learning

RL is a machine learning paradigm where an agent learns to make sequential decisions by interacting with an environment and receiving feedback through rewards or penalties. Unlike supervised learning, which relies on labeled data, RL employs a trial-and-error approach, making it highly adaptable to dynamic and uncertain environments. In RL, the primary objective is to maximize cumulative rewards defined by a reward function that encourages desirable actions and discourages unfavorable ones. RL agents can be broadly categorized as model-free or model-based. Model-free agents (DDPG, PPO, SAC) learn control policies directly from interactions with the environment without requiring a model of the environment’s dynamics, making them versatile for complex and unknown environments [12]. Model-based agents, on the other hand, use a learned or predefined model of the environment to predict future states and actions, allowing for more data-efficient learning but often at the expense of generalization in complex, stochastic settings. RL frameworks support agents functioning across discrete or continuous operational spectra. Discrete implementations, including Q-learning, excel in scenarios with limited action options such as chess moves or basic robotic commands [36]. Conversely, continuous-space architectures like DDPG, TD3, and SAC enable nuanced control essential for UAV maneuverability, where flight surfaces demand fine motor adjustments spanning infinite possibilities [8]. These agents allows viability for real-world systems, from multi-drone coordination to robotic manipulation, by improving training reliability and reducing computational overhead [37]. These agents prove particularly valuable for UAVs navigating unpredictable environments, where aerodynamic forces, sensor noise, and operational objectives create complex, non-linear interactions. Unlike model-dependent controllers, RL agents thrive in high-dimensional state spaces, autonomously discovering robust control laws [48].

### 4.2 Selection of RL agents

In RL, agents operate either in continuous or discrete action spaces. Discrete space agents are used in grid-based environments or games like chess, make decisions from a finite set of possible actions [49]. Conversely, continuous space agents operate in environments where actions vary smoothly and require precise control [50]. The control tasks in fixed wing UAVs includes managing roll, pitch, yaw, altitude, airspeed, and heading which are inherently continuous. The actuators of the UAV need precise continuous adjustments for UAV to maintain stability and performance. RL agents in continuous domain can produce precise control inputs necessary for these tasks thus making them an appropriate choice over discrete space agents. Among the myriad of continuous space RL algorithms, DDPG, TD3, PPO, TRPO, and SAC are selected for this study due to their advantages and proven track record in handling complex, high-dimensional control problems:

- **Deep Deterministic Policy Gradient (DDPG):** DDPG is a model-free, actor-critic algorithm that is designed for environments with continuous action spaces. By using deterministic policy updates and a replay buffer, it improves learning efficiency [24]. Its effectiveness for continuous control, particularly in fixed-wing UAV altitude-hold maneuvers, has been further demonstrated through comprehensive analyses of hybrid and unified control architectures, showing superior accuracy and response efficiency [51].
- **Twin Delayed Deep Deterministic Policy Gradient (TD3):** TD3 is based upon DDPG by addressing its limitations, including overestimation bias and training instability. In this algorithm, delayed policy updates and utilization of dual Q-networks are applied to stabilize the learning process [20].
- **Proximal Policy Optimization (PPO):** PPO is expressly chosen due to its application capabilities in getting a good balance between performance and computational cost. It enhances the conventional policy gradient approach with a clipped objective function that avoids large changes in a policy, helping to contribute to steady and consistent learning [25].
- **Trust Region Policy Optimization (TRPO):** TRPO has been designed to ensure monotonic improvement of the policy performance through the use of a trust region constraint on the policy updates, quantified through KL divergence [26].
- **Soft Actor-Critic (SAC):** SAC is unique because its framework is entropy-regularized, that is, it tends toward exploration through more stochastic policies. This characteristic is especially useful in scenarios with high uncertainty, including varying atmospheric conditions in UAV operations [27].

The selection of these five algorithms makes it possible to conduct a comprehensive comparison of their performance in UAV control. The unique properties of the algorithms, such as the exploratory model of SAC or the robustness of TD3, also offer important implications regarding the applicability of the algorithms for next-generation of UAV flight control systems.

Despite its potential advantages, such as offering finer control and suitability involving UAV dynamics, continuous-space RL has significant drawbacks. These include higher computational demands due to real-valued action sampling, increased sensitivity to noise in sensor data, and slower convergence in environments with sparse or delayed rewards. Additionally, the parameters in continuous-space policies often need fine-tuned reward shaping and optimization of hyperparameters to perform well, making it difficult to implement such policies in real-time systems. These RL-based control systems need to be designed keeping such constraints in mind when implemented on embedded UAV platforms.

### 4.3 Algorithm overview

RL-based flight controllers replace conventional PID loops with data-driven policies that can adapt, in real time, to non-linear dynamics and disturbances. The five algorithms considered, DDPG, TD3, PPO, TRPO, and SAC, employ an *actor* $\pi_{\theta }(a,|,s)$ that proposes continuous control actions and a *critic* $Q_{\phi }(s,a)$ that evaluates them. Training is guided by the Bellman expectation and deterministic policy gradients, with soft target-network updates used for stability in off-policy methods. These core elements are stated once here and referenced in the individual algorithm descriptions.

$$Q(s,a)=𝔼\;\left[r+\gamma \max_{a^{\prime }}Q(s^{\prime },a^{\prime })\right],$$ (15)

$$\nabla_{\theta }J={𝔼}_{s\sim D}\;\left[\nabla_{a}Q_{\phi }(s,a)\;{|}_{a=\pi_{\theta }(s)}\nabla_{\theta }\pi_{\theta }(s)\right],$$ (16)

$$\phi^{\prime }\leftarrow \tau \phi +(1-\tau )\phi^{\prime },\;\theta^{\prime }\leftarrow \tau \theta +(1-\tau )\theta^{\prime }.$$ (17)

These generic expressions form the foundation of the algorithm-specific variations discussed in the following subsections. Eq (17) defines the soft target update rule commonly used in off-policy methods like DDPG, TD3, and SAC to ensure stable learning.

In actor-critic architectures, the Q-function *Q*(*s*,*a*) estimates the expected cumulative reward (return) of taking an action *a* in a given state *s*. The policy $\pi_{\theta }(a\;|\;s)$ is trained to select actions that maximize these Q-values. This structure enables the agent to learn optimal behavior through continuous feedback, with the critic guiding the actor’s improvement based on estimated returns. These control signals, such as elevator or rudder deflections in UAVs, are directly output by the actor and executed in the flight system to adjust its dynamics in real time.

#### 4.3.1 Deep Deterministic Policy Gradient (DDPG).

**Fig 6:**
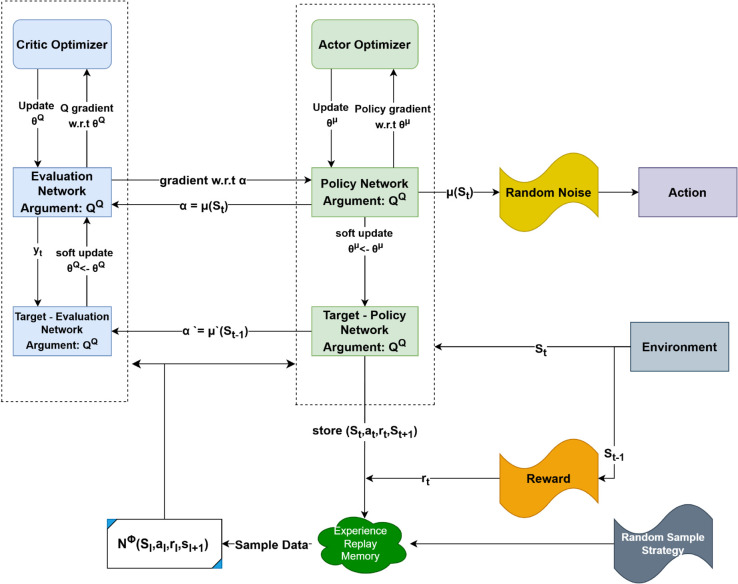
DDPG algorithm structure.

DDPG follows the generic actor-critic framework of Eqs (15)–(17) with deterministic actions and an off-policy replay buffer [19]. Its critic minimizes the TD error

$$L_{\text{DDPG}}(\phi )={𝔼}_{(s,a,r,s^{\prime })\;\sim \;D}\;\left[\;(Q_{\phi }(s,a)-y{)}^{2}\right],\;y=r+\gamma Q_{\phi^{\prime }}^{\prime }\;(s^{\prime },\pi_{\theta^{\prime }}^{\prime }(s^{\prime })),$$ (18)

while the actor is updated with the deterministic gradient in Eq (16). Fig 6 illustrates the architecture.

#### 4.3.2 Twin delayed deep deterministic policy gradient (TD3).

**Fig 7:**
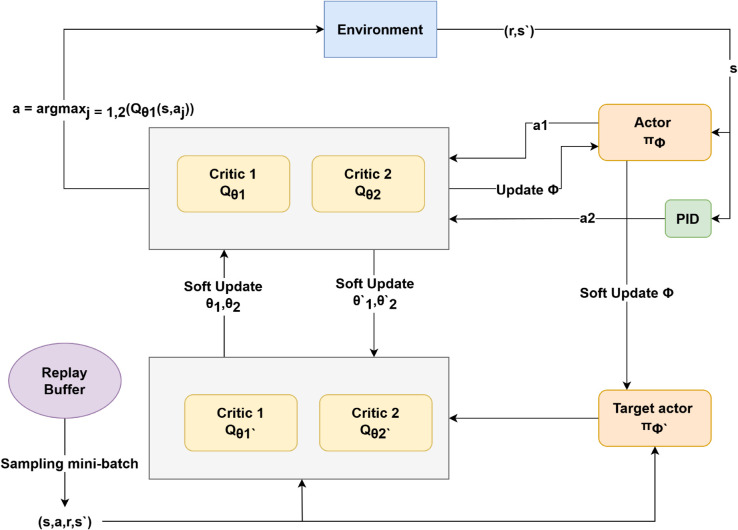
TD3 algorithm structure.

TD3 augments DDPG with three stabilizing ideas [20]: (i) Clipped double Q-learning uses two critics and the lower of their targets

$$y=r+\gamma \min_{i=1,2}Q_{\phi_{i}}^{\prime }\;(s^{\prime },\pi_{\theta^{\prime }}^{\prime }(s^{\prime })+\epsilon ),\;\epsilon \;\sim \;\text{clip}(N(0,\sigma ),-c,c);$$ (19)

(ii) Delayed policy updates: the actor (Eq 16) is updated every two critic steps; (iii) Target-policy smoothing: the clipped noise *ε* prevents over-fitting to sharp action peaks. The soft target update follows Eq (17). Fig 7 illustrates the TD3 workflow, including actor-critic updates and target policy smoothing.

#### 4.3.3 Proximal Policy Optimization (PPO).

PPO is an *on-policy* method that replaces the hard KL constraint of TRPO with a clipped surrogate loss [21]:

$$L_{\text{PPO}}(\theta )={𝔼}_{t}\;[\min\;(r_{t}(\theta ){\hat{A}}_{t},\;\text{clip}\;(r_{t}(\theta ),1-\epsilon ,1+\epsilon ){\hat{A}}_{t})],\;r_{t}(\theta )=\frac{\pi_{\theta }(a_{t}|s_{t})}{\pi_{\text{old}}(a_{t}|s_{t})}.$$ (20)

**Fig 8:**
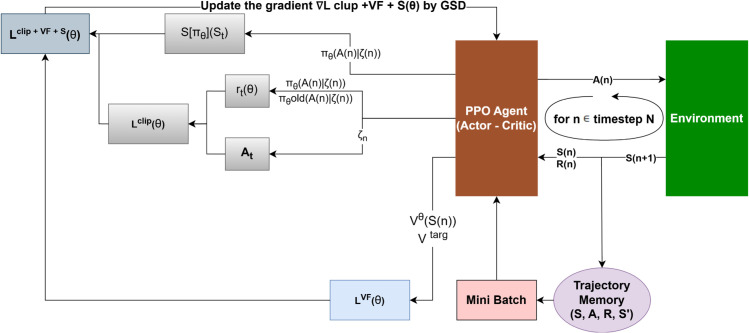
PPO algorithm structure.

Advantage estimates ${\hat{A}}_{t}$ use generalized advantage estimation (GAE), and an entropy bonus encourages exploration. Fig 8 shows the overall loop.

#### 4.3.4 Trust Region Policy Optimization (TRPO).

**Fig 9:**
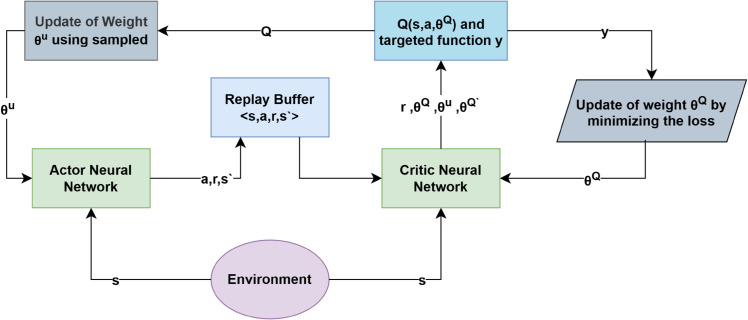
TRPO algorithm structure.

TRPO enforces a trust-region KL constraint explicitly:

$$\max_{\theta }\;{𝔼}_{(s,a)\sim \pi_{\text{old}}}\;\left[\frac{\pi_{\theta }(a|s)}{\pi_{\text{old}}(a|s)}\hat{A}(s,a)\right]\;\text{s.t.}\;{𝔼}_{s\sim \pi_{\text{old}}}\;[D_{KL}(\pi_{\text{old}}\;allel\;\pi_{\theta })]\leq \delta ,$$ (21)

which it solves with the natural-gradient step and conjugate-gradient search described in the original paper [22]. Fig 9 summarises the pipeline.

#### 4.3.5 Soft Actor-Critic (SAC).

**Fig 10:**
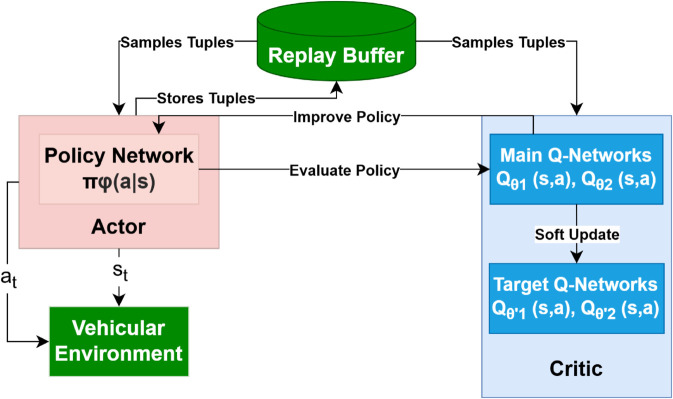
SAC algorithm structure.

SAC adds an entropy term αH(π) to favour stochastic, exploratory policies [23]. Its soft Bellman backup and actor loss are unique:

$$Q_{\phi }(s,a)\leftarrow r+\gamma \;{𝔼}_{s^{\prime }}\;[V_{\bar{\phi }}(s^{\prime })],\;V_{\bar{\phi }}(s^{\prime })={𝔼}_{a^{\prime }\;\sim \;\pi }\;[Q_{\bar{\phi }}(s^{\prime },a^{\prime })-\alpha \log\pi (a^{\prime }|s^{\prime })],$$ (22)

$$L_{\pi }(\theta )={𝔼}_{s\sim D}\;[\alpha \log\pi_{\theta }(a|s)-Q_{\phi }(s,a)],$$ (23)

with *α* tuned automatically to match a target entropy. Fig 10 depicts the flow.

#### 4.3.6 Strengths and limitations of RL agents.

Table 1 outlines the trade-offs associated with each RL algorithm. DDPG and TD3 excel in handling continuous action spaces but suffer from sensitivity to hyperparameters and convergence delays. PPO and TRPO offer stable learning, with TRPO providing stronger theoretical guarantees at the cost of computational overhead. SAC stands out for its robustness in uncertain environments due to entropy-driven exploration, though it requires more training data. Overall, each algorithm presents a unique balance between learning stability, computational cost, and control accuracy, making their suitability context-dependent for UAV applications.

**Table 1:** Strengths and limitations of RL algorithms.

Algorithm	Strengths	Limitations
**DDPG**	Continuous action handling, ideal for fine-tuned UAV control	Training instability due to sensitivity to hyperparameters.
	Model-free adaptation to non-linear dynamics.	Overestimation bias can lead to suboptimal policies.
**TD3**	Improved stability through delayed policy updates and clipped double Q-learning.	Slower convergence due to additional computational overhead.
	Reduces overestimation bias with dual critic networks.	Requires careful hyperparameter tuning.
**PPO**	Stable learning with controlled policy updates using a clipping mechanism.	Less conservative than TRPO, potentially unstable in highly dynamic environments.
	Computationally efficient, suitable for real-time UAV control.	Limited data efficiency in stochastic environments due to on-policy nature.
**TRPO**	Guaranteed policy stability with monotonic improvement.	High computational cost from trust region calculation.
	Handles non-linear dynamics effectively, suitable for precision tasks.	Slower convergence compared to PPO and TD3.
**SAC**	Encourages exploration with entropy maximization, leading to robust policies.	High computational demand due to dual optimization of reward and entropy.
	High stability and adaptability in uncertain environments.	Slower policy convergence requiring more training episodes.

### 4.4 Observation and action interface

The quality of reinforcement learning-based UAV systems is closely linked to how the agent perceives its environment (observations) and how it acts on it (actions). In this section, the state space representation and action space representation are described in terms of a structure that will remain the same throughout all the algorithms that will be used in this paper. The observation space provides the agent with a discrete set of measurements reflecting the UAV’s flight dynamics, while the action space dictates the range of the allowable range of control surface deflections the agent can apply. Together, they form the interaction loop, which defines the learning process by which the RL agent achieves precise and stable flight behavior.

#### 4.4.1 State space.

The state space in this UAV control problem gives RL agents significant and diverse information that they can use to control pitch, roll, and yaw. It consists of continuous variables representing the flight dynamics of the UAV that has the ability to model accurate control action on all axes [11]. These variables are explained in the following paragraphs.

1. **Pitch Control Parameters:** The state parameters used in controlling the pitch are necessary to deliver crucial information concerning the UAVs altitude, pitch orientation and the elevator deflection information allowing precise control of the altitude.
  $$S_{pitch}(t)=\left[e_{alt},{\dot{e}}_{alt},\int e_{alt},\theta ,\dot{\theta },\delta_{e}\right]$$
  The pitch control parameters are defined as
  - Altitude Error (*e*_*alt*_): Difference between commanded and measured altitude.
  - Altitude Rate (${\dot{e}}_{alt}$): Rate of change of altitude.
  - Pitch Angle (*θ*): Angle between the UAV’s longitudinal axis and the horizontal plane.
  - Pitch Rate ($\dot{\theta }$): Rate of change of pitch angle.
  - Integrated Altitude Error ($\int e_{alt}$): Cumulative altitude error over time.
  - Elevator Deflection ($\delta_{e}$): Current elevator deflection angle.
2. **Roll Control Parameters:** The roll control parameters ensure the UAV maintains lateral stability and tracks the desired roll angle effectively.
  $$S_{roll}(t)=\left[e_{\phi },\dot{\phi },\int e_{\phi },\delta_{a}\right]$$
  The roll control variables are defined as
  - Roll Angle Error ($e_{\phi }$): Difference between commanded and measured roll angle.
  - Roll Rate ($\dot{\phi }$): Rate of roll angle change.
  - Integrated Roll Error ($\int e_{\phi }$): Cumulative roll error over time.
  - Aileron Deflection ($\delta_{a}$): Current aileron position.
3. **Yaw Control Parameters:** The yaw control parameters focus on maintaining the UAV’s heading, ensuring stable directional control.
  $$S_{yaw}(t)=\left[e_{\psi },\dot{\psi },\int e_{\psi },\delta_{r}\right]$$
  The yaw control parameters are defined as
  - Yaw Angle Error ($e_{\psi }$): Difference between commanded and measured yaw angle (heading).
  - Yaw Rate ($\dot{\psi }$): Rate of yaw angle change.
  - Integrated Yaw Error ($\int e_{\psi }$): Cumulative yaw error over time.
  - Rudder Deflection ($\delta_{r}$): Current rudder position.

This structured state space guarantees that all needed information is supplied to the RL agents to control the flight dynamics of UAVs adaptively and provide stability as well as reach the desired goals [25,52].

#### 4.4.2 Action space.

The action space represents the set of possible control inputs that the RL agent can choose from at each time step to manage the UAV’s flight dynamics. In this context, the primary control actions involve adjusting the elevator, aileron, and rudder deflections to control altitude, airspeed, and heading [10].

- **Elevator Deflection ($\delta_{e}$):** The elevator controls the pitch of the UAV, directly influencing its altitude and airspeed. The action space for elevator deflection is continuous which allows the RL agent to apply any value within the specified range:
  $$A_{e}(t)=\delta_{e}\;\text{with limits}\;\delta_{e}\in [-30^{\circ },+30^{\circ }]$$
  This range represents the maximum upward or downward movement of the elevator.
- **Aileron Deflection ($\delta_{a}$):** Ailerons govern the roll motion of the UAV and play a key role in executing turns and maintaining lateral stability. The continuous action space corresponding to aileron deflection is:
  $$A_{a}(t)=\delta_{a}\;\text{with limits}\;\delta_{a}\in [-25^{\circ },+25^{\circ }]$$
  These bounds ensure accurate roll regulation while restricting undue lateral deviations.
- **Rudder Deflection ($\delta_{r}$):** The rudder governs the UAV’s heading by providing yaw control. Its deflection operates within a continuous action space:
  $$A_{r}(t)=\delta_{r}\;\text{with limits}\;\delta_{r}\in [-20^{\circ },+20^{\circ }]$$
  The chosen range allows the RL agent to manage directional variations while maintaining stability.

At each time step, the trained policy $\pi_{\theta }$ takes the current state *s*(*t*) and outputs control actions $a(t)=[\delta_{e},\delta_{a},\delta_{r}]$. These are sampled deterministically (DDPG, TD3) or stochastically (PPO, TRPO, SAC), depending on the algorithm. The continuous output ensures smooth adjustments in control surfaces, critical for flight stability.

### 4.5 Reward function

The reward function is designed to encourage the RL agent to minimize control errors and to maintain smooth control actions, making sure both stability and performance are achieved. The goal is to balance precision with efficiency by reducing error and minimizing control efforts.

#### 4.5.1 Dynamics of reward function.

The overall reward function that is used by the RL agent contains a weighted sum of the individual terms discussed below, it contains the need to minimize errors, to avoid large control inputs, and to ensure smooth and stable flight behavior.

The reward function *R* is given by the piecewise function described in Eq 24. The constants used in this formulation were selected empirically, following reward shaping principles to ensure stability, convergence, and consistent performance across different control axes. Specifically, ranges between [0.01,1] for tracking error penalties and [0.0001,0.01] for control effort penalties were explored during preliminary offline tuning to balance fast convergence with minimal actuator usage. These values were tuned to discourage overshoot, reduce aggressive control inputs, and ensure smooth responses across different flight regimes. Similar reward structures have also been employed in prior work, where terminal and intermediate step-based rewards are combined to promote both stable mission completion and incremental tracking accuracy [30].

$$R=\left\{ \begin{array}{cc} 0.1\cdot |5-e|-0.01\cdot |\Delta u|-|\dot{p}|+0.001\cdot I_{t} & \text{if }e<1\\ 0.05\cdot |1.5-e|-0.01\cdot |\Delta u|-|\dot{p}|+0.001\cdot I_{t} & \text{if }1\leq e\leq 5\\ -0.1\cdot |1.5-e|-0.01\cdot |\Delta u|-|\dot{p}|+0.001\cdot I_{t} & \text{if }e>5 \end{array}\right.$$ (24)

Where:

- *e* is the error (difference between observed and commanded control parameter), Δu is the change in deflection between time steps, $\dot{p}$ is the pitch/roll/yaw rate, *I*_*t*_ is the discrete time integrator value.

Additionally, if the termination criteria are met, the reward is given by:

$$R_{\text{termination}}=R-100$$ (25)

The specific values of these weights are tuned to balance precision and stability according to the requirements of the UAV control task.

- Error Minimization: Guarantees that the agent keeps the UAV close to the desired set point by penalizing deviations.
- Smooth Control: Ensures that there are no sudden changes in control surface deflection, resulting in more stable flight.
- Rate Penalty: Ensures there are no excessive pitch, roll, or yaw rates and makes the adjustments of the UAV trajectory smooth and regulated.
- Time Efficiency: Rewards the agent when it can reach stable flight quickly and reduces the corrective UAV motion.
- Termination Penalty: It penalizes actions that cause control loss or a critical deviation from the flight path.

In addition, the reward function has also been designed as a piecewise function which depends on the magnitude of the tracking error *e*. Under the proposed framework, the agent receives higher rewards for achieving finer control accuracy when the error is small. Whereas structured rewards or penalties are applied for moderate and large deviations. In particular, when *e* < 1, a higher reward coefficient is used to ensure precise tracking. In the case of moderate errors 1≤e≤5, the reward coefficient is reduced but still has a positive value for corrective actions. For large errors *e* > 5, the value of the reward coefficient is negative which penalizes unsafe or inefficient flight trajectories. This piecewise design provides a structure control approach that is in line with control objectives. The proposed design guarantees stability, responsiveness, and safety across changing flight conditions.

The inclusion of these constraints in the reward function ensures that the RL agent can achieve accurate and stable control of the UAV in terms of altitude, speed, and direction. The proposed piecewise formulation is specially useful for flight control under nonlinear deviations and helps in context-dependent learning.

## 5 Training and evaluation

The development and assessment of RL algorithms for UAV flight control consist of several stages. These encompass environment initialization, agent training, and subsequent performance evaluation. The overall aim is for each RL agent to acquire an optimal control policy capable of regulating the UAV’s control parameters.

### 5.1 Training process

To enhance the realism of training, UAV dynamics are simulated under practical external disturbances. The methodology is based on the interaction between RL agents and the UAV environment, through which appropriate rewards and penalties are defined. An iterative procedure is employed to progressively strengthen the agent’s control capability. The specific stages of the training process are summarized in Table 2.

**Table 2:** Training process for RL agents.

Step	Description
**Initialization**	Random policies and value functions are used to initialize each agent. The action space, state space, and reward function are predefined and the initial state of the UAV is randomized.
**State Observation**	The agent observes the state of the UAV, which included airspeed, altitude, pitch angle, pitch rate, and elevator deflection at every time step in order to take control decisions.
**Action Selection**	The agent selects an action (elevator deflection) from a continuous action space, allowing fine-grained adjustments within a bounded range to achieve control objectives.
**Environment Interaction**	The selected action is applied to the UAV model, and the environment updates the UAV’s state based on flight dynamics.
**Reward Calculation**	The agent receives a reward based on its performance, penalizing deviations from target values and encouraging smooth control actions to achieve steady flight conditions.
**Policy Update**	Using the observed state, action, reward, and new state, the agent updates its policy and value function. Off-policy methods (DDPG, TD3, SAC) use replay buffers, while on-policy methods (TRPO, PPO) use trajectory-based updates.
**Episode Termination**	Episodes end when the UAV reaches a time limit or when its state exceeds predefined safety thresholds, ensuring safe training conditions.
**Multiple Episodes**	The agent undergoes training across multiple episodes with varying initial conditions, refining its policy to improve control effectiveness over time.

The adopted training framework facilitates the optimization of UAV control in complex and dynamically changing environments. Within this framework, each RL agent is able to develop and refine its control policy to achieve optimal performance.

### 5.2 Evaluation criteria

Each RL agent was assessed across multiple dimensions to comprehensively evaluate its control capability. The evaluation criteria considered include convergence speed, control accuracy, stability, transient response, robustness against disturbances, policy generalization, computational and storage efficiency, and hyperparameter complexity. Collectively, these factors provide a holistic measure of the performance and robustness of RL agents in UAV stability and control. The detailed description of the evaluation metrics is provided in Table 3.

**Table 3:** Evaluation criteria for RL agents.

Evaluation Criteria	Description
**Convergence Speed**	The rate at which each agent learns an optimal policy. Faster convergence indicates fewer training episodes required to achieve satisfactory control.
**Control Accuracy**	Measured by the average absolute error (AAE) between actual and desired airspeed/altitude, reflecting how closely agents maintain target values.
**Stability of Control**	Refers to the smoothness of control actions, assessed by oscillation in elevator deflection and UAV state variables.
**Transient Response**	Measures how quickly and smoothly agents respond to changes in target values or disturbances, indicating responsiveness.
**Robustness to Disturbances**	Evaluates the agent’s ability to maintain control under various disturbance conditions, reflecting generalization capability.
**Policy Generalization**	Assesses how well agents apply learned policies across different flight scenarios, including steady-state flight, ascent/descent, and dynamic maneuvers.
**Computational Efficiency**	Evaluated based on the minimalism and smoothness of control actions, with excessive movements potentially increasing energy consumption.
**Storage Efficiency**	Amount of memory required during training. Off-policy methods (DDPG, TD3, SAC) require more memory due to replay buffers, whereas on-policy methods (PPO, TRPO) use less memory.
**Hyper-parameter Complexity**	Involves the complexity of adjusting hyperparameters. DDPG is easiest to tune, while PPO and TRPO are more challenging due to their extensive hyperparameter sets.

Each evaluation metric plays a crucial part in the comparative analysis of the RL agents’ capabilities for performance and adaptability in complex flight environments.

### 5.3 Implementation and training strategies

Each of the above agents has been implemented with specific architectures and hyperparameters. They have been designed to perform specific control tasks in continuous action spaces suitable for UAV operations. For all RL agents, sample time of 0.1 seconds is taken for Altitude and Roll Control. Whereas, for Heading Control a sample time of 0.01 seconds was used to ensure accurate tracking. The maximum number of steps agent could take in Roll control was 1300 steps, in Heading Control was 12000 steps and for Altitude Control agent could take a maximum of 2200 steps. The difference in steps were due to different simulation time and sample time due to precise control that could be attained by these agents.

#### 5.3.1 DDPG Implementation.

The DDPG agent was trained through iterative interaction with a simulated UAV environment to maintain its track using control surface deflections. Utilizing the Adam optimizer, the actor and critic networks were updated with learning rates of 10^−4^ and 10^−3^, respectively, with a discount factor (*γ*) of 0.99 to balance rewards. Target network smoothing ensured stability during learning. Training concluded once the agent achieved 80% of the theoretical maximum reward or attaining desired steps (1300 steps in Roll Control, 11000 steps in Heading Control and 2200 steps in Altitude Control) in an episodes indicating the agent’s proficiency in UAV control.

#### 5.3.2 TRPO implementation.

The TRPO agent was implemented to enhance stability in UAV control tasks through its trust region constraint, ensuring policy updates remain within safe bounds. The architecture includes a Gaussian actor network for generating actions and a critic network for value estimation, both employing fully connected layers with ReLU activations. The actor network features separate paths for the mean and standard deviation of the action distribution, promoting robust policy exploration. Training utilized the Adam optimizer with learning rates of 10^−4^ for the actor and 10^−3^ for the critic. The agent was configured with a discount factor (*γ*) of 0.99, an experience horizon of 512, and a KL divergence limit of 0.02 to ensure policy stability. Agents were trained until they achieved 80% of the theoretical maximum reward and only the best agents with the most stable performance were selected for further analysis.

#### 5.3.3 PPO implementation.

PPO was used to improve the stability and efficiency of the UAV control process by limiting the update of the policy within a clipped range, avoiding a large deviation in training. The architecture is a Gaussian actor network that generates actions and a critic network that estimates values and consists of fully connected layers with ReLU activation functions. The actor network’s output paths for the mean and standard deviation of the action distribution ensured a balance between exploration and exploitation. The training made use of the Adam optimizer, with learning rates of 10^−4^ for the actor and 10^−3^ for the critic, and a discount factor (*γ*) of 0.997. The agent was trained with an experience horizon of 1024 and a clipping factor of 0.04. The training stopped when the agent achieved 80% of the theoretically possible maximum reward.

#### 5.3.4 TD3 implementation.

TD3 has been implemented using two critic networks and delaying policy updates to address overestimation bias and enhance stability in UAV control tasks. The actor network has been used for the generation of deterministic actions, whereas the dual critic networks estimate the value of the actions for robustness. Both networks utilize fully connected layers with ReLU activations. The training employed the Adam optimizer with learning rates of 10^−4^ and 10^−3^ for the actor and the critics, respectively. Moreover, a discount factor (*γ*) of 0.99 and target smoothing are used to stabilize the policy updates. The agent has been trained until it achieved 80% of the theoretically possible maximum reward. The limit has been set to ensure effective performance under dynamic flight conditions.

#### 5.3.5 SAC implementation.

The SAC agent implementation has been carried out for continuous control of UAV operations. In SAC implementation, the stability and robustness have been achieved through an entropy-regularized framework. The implementation employed stochastic actor network and dual critic networks for increased exploration and reduced overestimation bias. The actor sampled actions with a Gaussian policy, whereas the critics assessed the actions to improve the policy. The training has been conducted in a UAV simulation involving iterative interactions using the Adam optimizer with learning rates of 10^−4^ and 10^−3^ for the actor and critic, respectively. The discount factor (*γ*) is taken as 0.99. The training was carried out until the agent acquired 80% of the theoretical maximum reward. This limit has been set to achieve control proficiency and stability for further analysis.

#### 5.3.6 List of key hyperparameters across different RL agents.

A detailed description and parameter values for different RL approaches are provided in Table 4. The Table 4 summarizes the key hyperparameter settings used in the flight control missions by all the agents. The values have been presented side by side to analyze their impacts on the performance and stability of UAV control systems. An in-depth look at Table 4 reveals how different parameters can be compared for achieving optimal control performance under different scenarios.

**Table 4:** Hyperparameters for all agents.

Hyperparameter	DDPG	PPO	TRPO	SAC	TD3	Description
**Actor Learning Rate**	10^−4^					Rate at which the actor network weights are updated during training.
**Critic Learning Rate**	10^−3^					Rate at which the critic network weights are updated.
**Discount Factor (*γ*)**	0.99					Balances short-term and long-term rewards.
**Batch Size**	64					Number of samples used in each training batch.
**Experience Buffer Size**	10^6^					Capacity of the replay buffer used to store past experiences for off-policy learning.
**Optimizer**	Adam					Optimization algorithm used for training the networks.
**Use Device**	GPU					Utilizes GPU to accelerate training
**Target Smoothing Factor**	10^−3^					Factor used to update the target networks for stability.
**Noise Variance**	0.3					Initial variance of noise added to actions for exploration.
**Noise Variance Decay Rate**	10^−5^					Rate at which the exploration noise variance decays over time.
**Gradient Threshold**	1					Maximum allowable gradient to prevent exploding gradients.
**L2 Regularization Factor**	$2\times 10^{-4}$					Regularization factor applied to prevent overfitting in the critic network.
**MiniBatch Size**			128		64	Number of samples per mini-batch during training.
**Experience Horizon**		1024	512			Number of time steps per training episode.
**Entropy Loss Weight**		0.1	0.95			Weight for the entropy term to encourage exploration.
**KL Divergence Limit**			0.02			Maximum allowed KL divergence to constrain policy updates.
**Number of Conjugate Gradient Iterations**			10			Number of iterations for the conjugate gradient method in policy optimization.
**Number of Line Search Iterations**			10			Number of iterations for the line search to find the optimal policy update step size.
**Conjugate Gradient Damping**			0.01			Damping factor for the conjugate gradient to stabilize computations.
**Conjugate Gradient Residual Tolerance**			10^−8^			Tolerance for the residual in the conjugate gradient method.
**GAE Factor**		0.5	0.95			Generalized Advantage Estimation (GAE) factor for advantage computation.
**Number of Epochs**			3			Number of epochs for policy updates in each training cycle.
**Clipping Factor**		0.04				Maximum allowed policy update to prevent large changes.
**Entropy Loss Weight**		0.1				Weight for the entropy term to encourage exploration.
**Number of Epochs**		3				Number of epochs for policy updates in each training cycle.
**Advantage Estimate Method**		GAE				Method used to estimate advantage, balancing bias and variance.
**Target Smoothing Factor**				10^−3^		Smoothing factor for updating target networks to stabilize learning.
**Num Warm Start Steps**				1000		Initial steps taken without learning to populate the replay buffer.
**Entropy Coefficient (*α*)**				Adaptive		Balances exploration and exploitation by adjusting the randomness in actions.

## 6 Results and discussion

Different RL agents have been trained in the simulated UAV control environment. The control objectives mainly focused on heading, roll, and altitude control. The resulting performance metrics have been used to compare the effectiveness of each agent: TRPO, TD3, SAC, PPO, DDPG, and a classic PID controller. The following evaluation focuses on the relative performance of RL agents and a conventional PID baseline under various flight conditions. SAC and TD3 exhibited exceptional robustness to disturbances and achieved smoother control actions with minimal oscillations. TRPO and PPO showed reliable stability during steady-state conditions but required more training episodes for convergence. DDPG, while faster in convergence, was more sensitive to environmental changes. The performance metrics for heading control, roll control, and altitude control across all agents and PID are discussed below, providing a detailed comparison of their effectiveness in dynamic UAV environments.

### 6.1 DDPG training result

**Fig 11:**
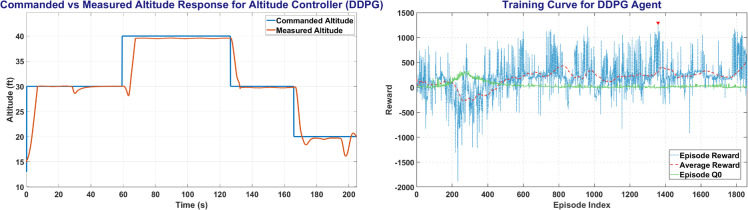
The Altitude Control (left) and Training Curve (right) obtained on DDPG agent.

DDPG Agent was trained for Altitude, Heading and Roll Control of UAV with a step size of 0.1, 0.01 and 0.1 seconds respectively over more than 300,000 steps. Fig 11 shows the DDPG training window and the plot between the commanded altitude and states. The left side plot of Fig 11 shows the Commanded Altitude vs Observed Altitude, where Red line shows State of the UAV and Blue line shows the commanded altitude. The right side of Fig 11 shows the training window plot. DDPG achieved a steady state error of less than 5% in less than 10 seconds with a peak average reward attained in less than 1400 episodes.

**Fig 12:**
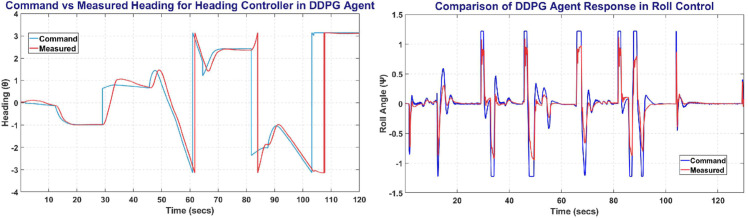
The Heading Control (left) and Roll Control (right) obtained on DDPG agent.

The Fig 12 represents the Heading and Roll Controller response with the left plot showing heading angle (*ψ*) commanded and measured. Whereas the right Fig shows the Roll angle (*ϕ*) commanded and measured, and its variation with respect to time.

### 6.2 TRPO training result

**Fig 13:**
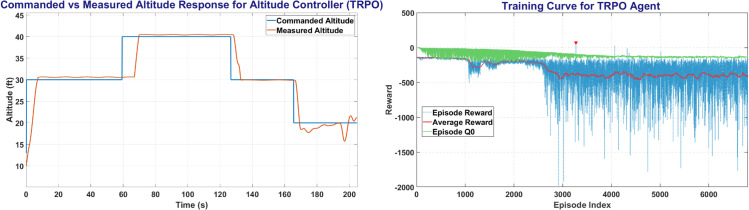
The Altitude Control (left) and Training Curve (right) obtained on TRPO agent.

TRPO was trained on MATLAB environment for all 3 main controllers over more than 400,000 steps. Fig 13 shows the TRPO training window and the plot between the commanded altitude and states. The left side of Fig 13 shows the Commanded Altitude vs Observed Altitude, where Red line shows State of the UAV and Blue line shows the commanded altitude. The right side of Fig 13 shows the training window plot. TRPO achieved a steady state error of less than 5% in less than 10 seconds with a peak average reward attained in slightly over 3200 episodes.

**Fig 14:**
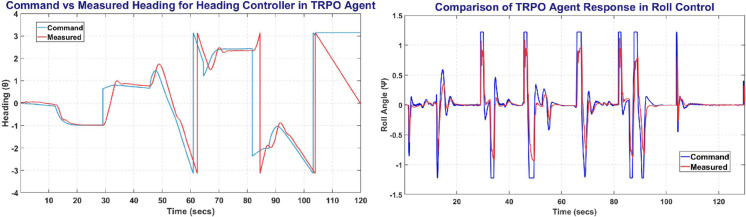
The Heading Control (left) and Roll Control (right) obtained on TRPO agent.

The Fig 14 represents the Heading and Roll Controller response with the left plot showing heading angle (*ψ*) commanded and measured. Whereas the right figure shows the Roll angle (*ϕ*) commanded and measured, and its variation with respect to time.

### 6.3 PPO training result

**Fig 15:**
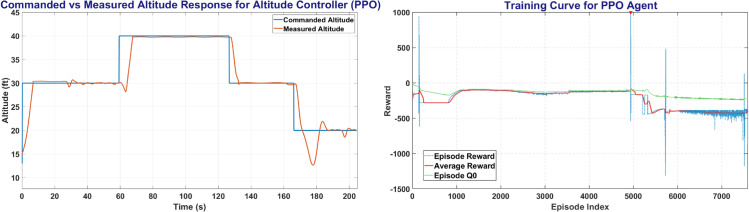
The Altitude Control (left) and Training Curve (right) obtained on PPO agent.

PPO was trained on MATLAB environment for all controllers with more than 800,000 total steps. Fig 15 shows the PPO training window and the plot between the commanded altitude and states. The left side of Fig 15 shows the Commanded Altitude vs Observed Altitude, where Red line shows State of the UAV and Blue line shows the commanded altitude. The right side of Fig 15 shows the training window plot. PPO achieved a steady state error of less than 5% in less than 10 seconds but had slight oscillations in between simulations, it completed its training with a peak average reward attained at 3500 episodes.

**Fig 16:**
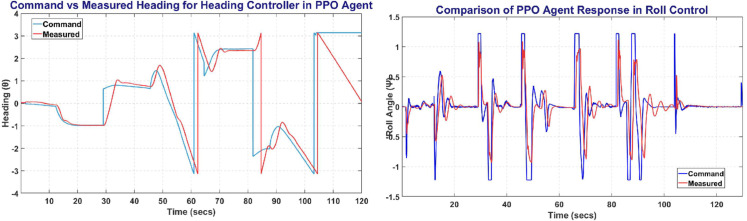
The Heading Control (left) and Roll Control (right) obtained on PPO agent.

The Fig 16 represents the Heading and Roll Controller response with the left plot showing heading angle (*ψ*) commanded and measured. Whereas the right figure shows the Roll angle (*ϕ*) commanded and measured, and its variation with respect to time.

### 6.4 TD3 training result

**Fig 17:**
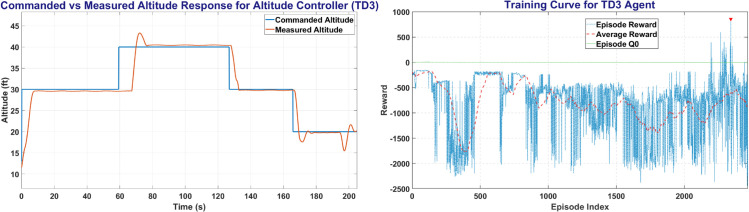
The Altitude Control (left) and Training Curve (right) obtained on TD3 agent.

TD3 was trained on MATLAB environment with a total of more than 500,000 steps for UAV control. Fig 17 shows the TD3 training window and the plot between the commanded altitude and states. The left side of Fig 17 shows the Commanded Altitude vs Observed Altitude, where Red line shows State of the UAV and Blue line shows the commanded altitude. The right side of Fig 17 shows the training window plot. TD3 achieved a steady state error of less than 5% in less than 10 seconds with a peak average reward attained in less than 2500 episodes.

**Fig 18:**
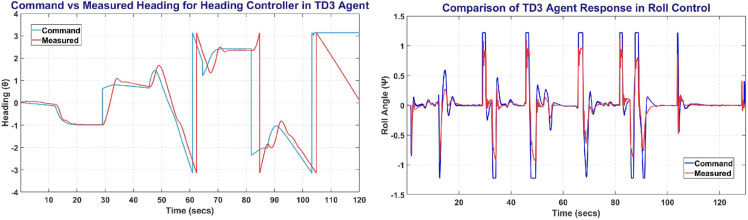
The Heading Control (left) and Roll Control (right) obtained on TD3 agent.

The Fig 18 represents the Heading and Roll Controller response with the left plot showing heading angle (*ψ*) commanded and measured. Whereas the right figure shows the Roll angle (*ϕ*) commanded and measured, and its variation with respect to time.

### 6.5 SAC training result

**Fig 19:**
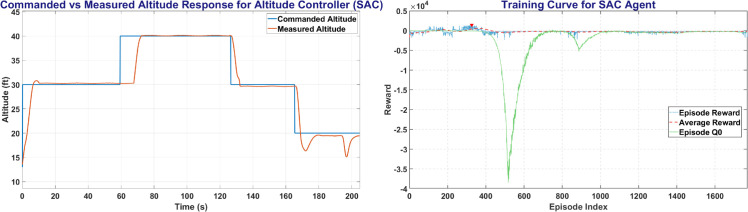
The Altitude Control (left) and Training Curve (right) obtained on SAC agent.

SAC was trained on MATLAB environment with 140,000 steps for all UAV control parameters. Fig 19 shows the SAC training window and the plot between the commanded altitude and states. The left side of Fig 19 shows the Commanded Altitude vs Observed Altitude, where Red line shows State of the UAV and Blue line shows the commanded altitude. The right side of Fig 19 shows the training window plot. SAC performed the best, it achieved a steady state error of less than 5% in less than 10 seconds with a peak average reward attained in less than 400 episodes.

**Fig 20:**
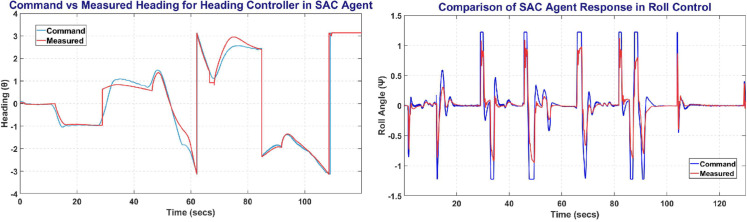
The Heading Control (left) and Roll Control (right) obtained on SAC agent.

The Fig 20 represents the Heading and Roll Controller response with left plot showing heading angle (*ψ*) commanded and measured. Whereas the right figure shows the Roll angle (*ϕ*) commanded and measured, and its variation with respect to time.

### 6.6 PID result

**Fig 21:**
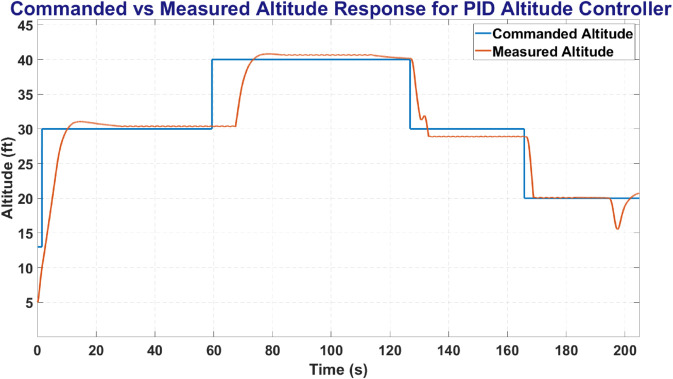
The altitude control for PID controller.

PID was tuned using Open Loop Transfer Function to find relevant gains for individual controllers for best control accuracy. Fig 21 shows the PID plot between the commanded altitude and states, where Red line shows State of the UAV and Blue line shows the commanded altitude. PID attained a steady state error of 6% with minimal overshoot and undershoot.

**Fig 22:**
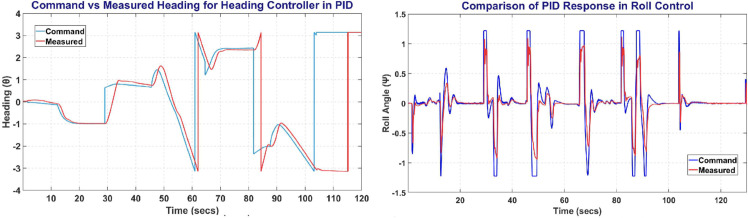
The Heading Control (left) and Roll Control (right) of PID controller.

The Fig 22 represents the Heading and Roll Controller response with left plot showing heading angle (*ψ*) commanded and measured. Whereas the right figure shows the Roll angle (*ϕ*) commanded and measured, and its variation with respect to time.

### 6.7 Control surface deflections

**Fig 23:**
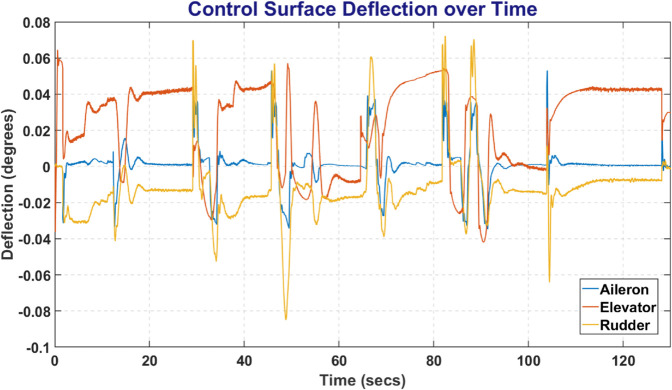
The deflection of control surfaces over time.

All RL Agents and PID controller were able to maintain the track of fixed wing UAV with adjusting the control surface deflection such that it remains in its bounded limits and also on track. The Fig 23 represents the deflection of Aileron, Elevator and Rudder over time to maintain a UAV on a track. Since there was minimal difference between error deflection, so, the figure represents the average deflection of control surface.

### 6.8 Comparative results

**Fig 24:**
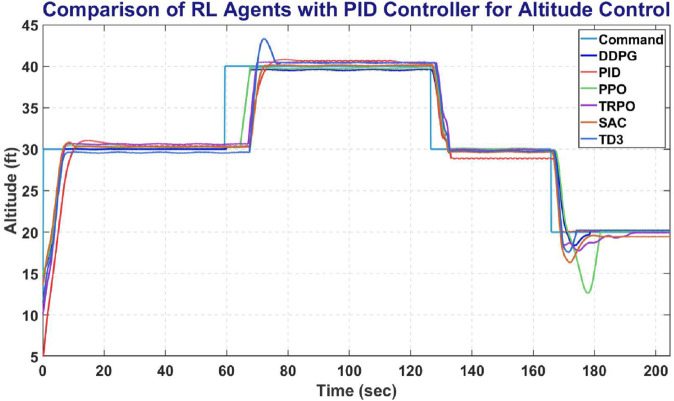
Comparison of RL agent and PID response for altitude controller.

This section provides a comparative overlay of all five RL agents and the PID controller, analyzing their performance in altitude, heading, and roll control tasks. Fig 24 represents the comparison of Agents with a Tuned PID Controller along with the reference Altitude, SAC outperforms its competitors with minimal overshoot and undershoot along with early settling time and low steady state error.

**Fig 25:**
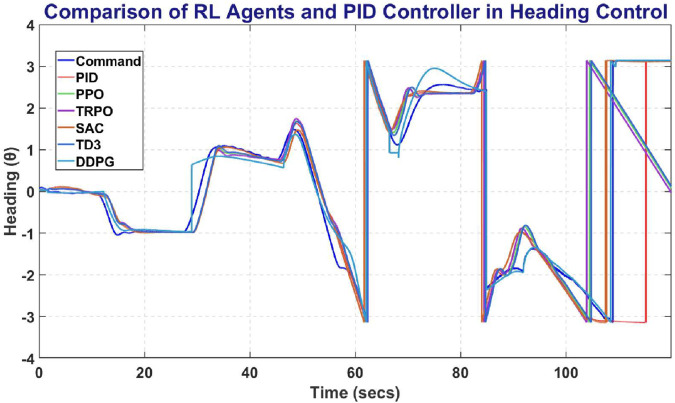
Comparison of RL agent and PID response for heading controller.

Fig 25 represents the comparative analysis on Heading Control problem. SAC outperforms with maintaining heading closest to Commanded Heading.

**Fig 26:**
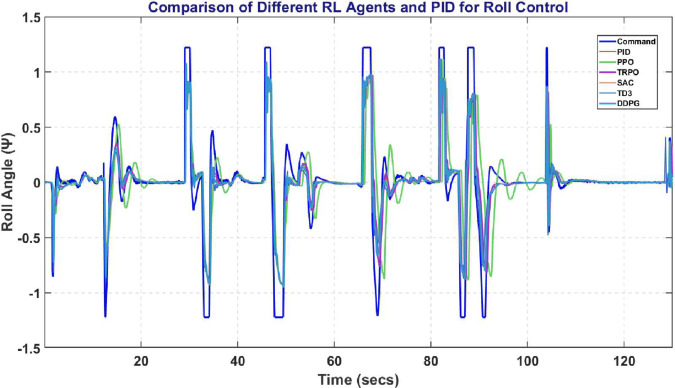
Comparison of RL agent and PID response for roll controller.

Fig 26 represents the comparative analysis on maintaining Roll Angle with SAC outperforming all the competitors with minimal error and a smooth transient response.

## 7 Comparison of RL agents

The RL agents, DDPG, TD3, SAC, TRPO, and PPO, were trained in the UAV simulation environment, with SAC showing the best performance in terms of convergence speed and control accuracy. TD3 also demonstrated strong stability, while DDPG, TRPO, and PPO offered varying levels of efficiency and robustness.

### 7.1 Performance evaluation

To assess performance of the RL algorithms, a comprehensive comparison is made based on key criteria mentioned in Table 3.

The performance of the RL agents in flight control is assessed across several dimensions. Convergence Speed categorizes agents into fast, moderate, and slow based on the number of episodes required to reach peak performance, with SAC generally being the fastest. When evaluating control accuracy, SAC consistently demonstrated higher precision compared to the other algorithms, whereas PPO occasionally produced oscillatory responses. In terms of stability, both SAC and TRPO yielded smoother trajectories with reduced oscillations, highlighting their suitability for applications requiring consistent control. With respect to robustness, SAC maintained reliable performance under environmental disturbances, surpassing PPO and DDPG in this regard. For policy generalization, SAC and TD3 adapted more effectively to different flight scenarios, indicating stronger transferability. Computational efficiency, however, was more favorable in PPO and TD3, since SAC and TRPO involved greater computational cost due to their algorithmic complexity. From a storage perspective, PPO and TRPO required comparatively less memory, whereas SAC demanded more storage resources. Finally, regarding the complexity of implementation, DDPG was relatively straightforward to configure with fewer hyperparameters, while TRPO required extensive tuning and was the most demanding in terms of parameter sensitivity.

The Table 5 serves as a consolidated summary of performance metrics across all five RL agents, providing a clear side-by-side comparison of their capabilities in terms of convergence, control accuracy, robustness, computational efficiency, and hyperparameter complexity. SAC shows superior control accuracy, achieving a steady-state error of less than 6% in less than 10 seconds with peak performance in fewer than 400 episodes, making it highly robust and stable. TD3 and DDPG reached optimal performance in less than 2500 and 1400 episodes, respectively, while PPO and TRPO required more episodes (3500 and 3200) but offered stability. The transient response of all the agents was similar, because of their same sample time. Robustness ratings, as reported in the table, are based on empirical evaluations under variable mission profiles and wind disturbances, with performance averaged across multiple randomized trials to reflect consistent behavior under uncertainty.

**Table 5:** Comparison of RL agents.

Criteria	DDPG	TD3	SAC	TRPO	PPO
**Convergence Speed**	< 1000 Episodes	1000-2500 Episodes	< 1000 Episodes	1000-2500 Episodes	>2500 Episodes
**Control Accuracy**	<±4% Error	<±4% Error	<±3% Error	<±4% Error	<±5% Error
**Stability of Control**	< 10% Oscillations	< 7% Oscillations	< 7% Oscillations	< 12% Oscillations	< 12% Oscillations
**Robustness to Disturbances**	Handles > 95% Disturbances	Handles > 90% Disturbances	Handles > 95% Disturbances	Handles > 85% Disturbances	Handles > 90% Disturbances
**Policy Generalization (with Random Start Position)**	Moderate Generalization	High Generalization	Very High Generalization	High Generalization	High Generalization
**Computational Efficiency**	Moderately Efficient	Resource Intensive	Low Computational Overhead	Low Computational Overhead	Resource Intensive
**Transient Response**	< 5 Seconds	< 5 Seconds	< 5 Seconds	< 5 Seconds	< 5 Seconds
**Storage Efficiency**	Moderate Memory Usage	Moderate Memory Usage	Low Memory Usage	High Memory Usage	High Memory Usage
**Hyper-parameter Complexity**	12 Hyperparameters	13 Hyperparameters	12 Hyperparameters	15 Hyperparameters	13 Hyperparameters

### 7.2 Training time and hardware details

All training experiments were conducted on a high-performance workstation equipped with the following specifications: dual Intel^®^ Xeon^®^ Platinum 8173M CPUs operating at 2.00 GHz, 128 GB DDR4 RAM, and an NVIDIA^®^ GeForce RTX 4090 GPU with 24 GB of dedicated memory. The system ran Windows 11 Pro (64-bit) with MATLAB R2024a, Simulink, and the Reinforcement Learning Toolbox installed. A 512 GB NVMe solid-state drive (SSD) was used for local data storage and simulation caching.

Each reinforcement learning agent was trained independently using consistent initial seeds, identical simulation parameters (including episode length, termination conditions, and reward structure), and equivalent environmental conditions. Where supported, training was accelerated using GPU-based computation and batch replay buffers were stored in RAM to minimize I/O latency. These configurations enabled efficient policy learning while preserving training consistency across all evaluated agents.

As shown in Table 6, SAC and TD3 achieved the fastest per-step training performance due to efficient use of GPU acceleration and off-policy learning. In contrast, DDPG and TRPO exhibited the longest time per step, likely due to larger network sizes or more complex optimization dynamics. PPO demonstrated a good trade-off between overall training time and convergence efficiency.

**Table 6:** Training time, total steps, and step efficiency of RL agents.

Agent	Training Time (HH:MM:SS)	Total Steps	Time per Step (s)
DDPG	66:26:45	1,378,258	0.1735
PPO	34:29:07	1,308,872	0.0948
SAC	41:28:56	2,362,178	0.0638
TD3	45:26:27	2,483,716	0.0659
TRPO	40:56:14	838,064	0.1758

Importantly, the training times reported here reflect the duration required for each algorithm to reach convergence, defined as the point where average episodic return stabilized and performance no longer improved significantly across successive episodes. This metric provides a meaningful comparison of the computational cost associated with the training of each RL agent with its effective policy.

### 7.3 Discussion

The comparison presented in Table 5 indicates that SAC emerges as the most robust and accurate algorithm, particularly excelling in environments characterized by disturbances and uncertainty. However, it is also the most computationally expensive. TD3 strikes a balance between convergence speed, stability, and robustness, making it a suitable choice for dynamic control problems where computational resources are limited. PPO is computationally cheaper and easier to achieve fast convergence and stable learning, making it suitable for real-time applications, although it lacks the precision and robustness of SAC or TD3 in complex scenarios. DDPG and TRPO each demonstrate strengths in particular areas, however, DDPG suffers from limited stability, while TRPO is computationally intensive. This comparative analysis highlights the trade-offs between different RL algorithms, helping to select a suitable agent based on specific requirements such as precision, stability, and computational constraints for UAV flight control tasks.

## 8 Comparison of PID and RL

The comparison in this study is not intended to equate RL and PID in terms of control theory complexity, but rather to highlight the practical differences in performance when applied to the same UAV task. RL controllers were evaluated in a non-linear, multivariable environment where PID controllers were manually tuned for each axis, which reflects their conventional use in cascaded SISO structures within UAV autopilot systems.

### 8.1 Summary table: Comparison of RL agents and PID controllers

The Table 7 compares the most robust RL agent, SAC with PID controller based on evaluation parameters listed below.

**Table 7:** Comparison of PID and RL agents.

Criteria	SAC	PID
**Convergence Speed**	< 1000 Episodes	No Training
**Control Accuracy**	<±3% Error	<±10% Error
**Stability**	<7% Oscillations	<10% Oscillations
**Robustness to Disturbances**	Handles >95% Disturbances	Handles >95% Disturbances
**Policy Generalization**	Very High Generalization	No Generalization
**Computational Efficiency**	Low Computational Overhead	Low Computational Overhead
**Storage Efficiency**	Low Memory Usage	No Memory Usage
**Hyperparameter Complexity**	12 Hyperparameters	3 Tuning Parameters

The SAC agent significantly outperforms PID controllers in terms of control accuracy, robustness, and adaptability, especially in complex and dynamic environments. It maintains stable flight and responds effectively to disturbances, though these advantages come at the cost of increased computational demands, longer training times, and more complex hyperparameter tuning. In contrast, PID controllers offer a simple and computationally efficient solution for linear and fixed environments. While they can deliver effective control once properly tuned, they lack adaptability and require frequent re-tuning to handle new or varying flight conditions.

## 9 Conclusion

This study involved the implementation and evaluation of state-of-the-art reinforcement learning algorithms, DDPG, TD3, SAC, PPO, and TRPO, in the control of a fixed wing UAV, particularly in managing airspeed, altitude, roll, and heading. Each algorithm was assessed for its effectiveness in controlling the elevator, aileron, and rudder which are critical control surfaces required to maintain stable UAV flight. The agents were analyzed in terms of training speed, robustness, adaptability, and memory efficiency.

The findings highlight that RL-based controllers provide clear advantages over conventional approaches, such as PID, particularly in handling the UAV’s nonlinear behavior and enabling smoother control in dynamic environments. The results showed that DDPG is the simplest and fastest algorithm to tune for UAV applications where stability requirements are modest. In contrast, TD3 and SAC offered greater robustness than DDPG but required longer training. PPO and TRPO demonstrated stronger resilience and stability, though at the cost of increased training time and hyperparameter tuning. Overall, off-policy methods such as TD3 and SAC achieve a practical balance among speed, stability, and memory efficiency, making them well-suited for UAV control. On the other hand, on-policy methods like PPO and TRPO may yield superior resilience but their complexity and resource demands reduce feasibility for real-time deployment.

Relative to classical PID controllers, RL-based controllers exhibit substantial benefits by managing UAV nonlinear dynamics more effectively and ensuring smoother performance in dynamic environments. In parallel, work is underway to transition this framework toward HIL testing with real-time onboard inference, laying the groundwork for future real-world deployment.

## Supporting information

S1 Aero_Data Aerodynamic data of UAV. (XLSX)

S2 Agents_Data Hyperparameters details for algorithms. (XLSX)

S3 Source_Code GitHub repository link. (DOCX)
