# Supplementary material for: Reinforcement learning for UAV flight controls: Evaluating continuous space reinforcement learning algorithms for fixed-wing UAVs
Source: PLoS One. 2025 Oct 9;20(10):e0334219. doi: 10.1371/journal.pone.0334219 (PMC12510717; doi:10.1371/journal.pone.0334219)
Supplement: S3 Source_Code — (DOCX) [file pone.0334219.s003.docx]

This file provides the GitHub repository link for the source code:

<https://github.com/HasanRazaKz/Comparative-Evaluation-of-RL-in-FC/releases/tag/RL>
